# A prospective study of treatment of carbapenem-resistant *Enterobacteriaceae* infections and risk factors associated with outcome

**DOI:** 10.1186/s12879-016-1979-z

**Published:** 2016-11-03

**Authors:** Claudia M. D. de Maio Carrilho, Larissa Marques de Oliveira, Juliana Gaudereto, Jamile S. Perozin, Mariana Ragassi Urbano, Carlos H. Camargo, Cintia M. C. Grion, Anna Sara S. Levin, Silvia F. Costa

**Affiliations:** 1 Internal Medicine Department, Londrina State University, Paraná, Brazil; 2 Microbiologist, Londrina, Brazil; 3 Postgraduate student, Londrina State University, Paraná, Brazil; 4 Statistics Department, Londrina State University, Paraná, Brazil; 5 Instituto Adolfo Lutz, Centro de Bacteriologia, São Paulo, Brazil; 6 Infectious Diseases Department, University of State of São Paulo, São Paulo, Brazil; 7 Infectious Diseases Department of Medicine School of University of São Paulo, Avenida Doutor Enéas de Carvalho Aguiar, 470, São Paulo, SP 05403-000 Brazil

**Keywords:** *Enterobacteriaceae*, Drug resistance, Multiple, Bacterial, Carbapenems, Colistin

## Abstract

**Background:**

To describe the clinical and microbiological data of carbapenem-resistant *Enterobacteriaceae* (CRE) infections, the treatment used, hospital- and infection-related mortality, and risk factors for death.

**Methods:**

A prospective cohort conducted from March 2011 to December 2012. Clinical, demographic, and microbiological data such as in vitro sensitivity, clonality, carbapenemase gene mortality related to infection, and overall mortality were evaluated. Data were analyzed using Epi Info version 7.0 (CDC, Atlanta, GA, USA) and SPSS (Chicago, IL, USA).

**Results:**

One hundred and twenty-seven patients were evaluated. Pneumonia, 52 (42 %), and urinary tract infections (UTI), 51 (40.2 %), were the most frequent sites of infection. The isolates were polyclonal; the *Bla*_KPC_ gene was found in 75.6 % of isolates, and 27 % of isolates were resistant to colistin. Mortality related to infection was 34.6 %, and was higher among patients with pneumonia (61.4 %). Combination therapy was used in 98 (77.2 %), and monotherapy in 22.8 %; 96.5 % of them were UTI patients. Shock, age, and dialysis were independent risk factors for death. There was no difference in infection-related death comparing colistin-susceptible and colistin-resistant infections (*p* = 0.46); neither in survival rate comparing the use of combination therapy with two drugs or more than two drugs (*p* = 0.32).

**Conclusions:**

CRE infection mortality was higher among patients with pneumonia. Infections caused by colistin-resistant isolates did not increase mortality. The use of more than two drugs on combination therapy did not show a protective effect on outcome. The isolates were polyclonal, and the *bla*_KPC_ gene was the only carbapenemase found. Shock, dialysis, and age over 60 years were independent risk factors for death.

## Background

Carbapenem-resistant *Enterobacteriaceae* (CRE), and in particular, carbapenemase-producing KPC type have become globally endemic [1–3]. CRE resistance to colistin has been reported in several countries in the last years [4–7] and infections caused by these bacteria are associated with high mortality [8–10]. KPC is the most frequent carbapenemase described in Enterobacteriaceae in Brazil with great mortality [8, 11]. One cohort study in Brazil described 118 patients with CRE infections in a period of almost four years. The authors reported catheter-related bloodstream infections as the most frequent among these patients resulting in overall 30-day mortality rate of 45 % [11].

Risk factors associated with death in CRE infections are those related to the seriousness of patients’ conditions and infections, such as the APACHE and SOFA scores, systemic infections, and sepsis [4, 6, 8, 9]. Although, colistin resistance has been associated with high mortality, there is controversy about the impact of resistance to colistin on prognosis [9, 10, 12–15]. Combination therapy has been advocated as the treatment of choice to treat systemic infection, mainly bloodstream infection caused by CRE [12–15]; however, the impact of such strategy on treatment of other types of infection, such as pneumonia and urinary tract infection, and the benefit of combination therapy with more than two drugs need to be better addressed.

Thus, the knowledge of risk factors for death in infections such as those caused by CRE could be useful in directing therapeutic resources in high-risk patients and planning intervention in preventable factors that can reduce mortality.

The objectives of this study were to describe the clinical and microbiological characteristics of CRE infections, including pneumonia susceptible and resistant to colistin, the treatment regimens used, infection-related mortality, and risk factors associated with death.

## Methods

### Study population and design

A prospective cohort was conducted at the 317-bed University Hospital of Londrina (Paraná, Brazil), from March 2011 to December 2012. This is a public university hospital, with ten Intensive Care Units (ICU) beds, ten Burn unit beds, that serving a geographic region with an estimated population of 1,790,000 inhabitants. Adult patients (≥18 years) with documented infection by CRE were accompanied. The Ethics Committee of the State University of Londrina and the Medical School of the University of São Paulo approved the study. All patients or their legal representatives filled out a form agreeing to participate in this study, which was approved (number 0318/12) by the ethics committee of both hospitals.

### Data collection and definitions

#### Data collection

Data were collected and analyzed from patient’s charts and the electronic hospital database.

#### Case definition

Hospitalized patients treating infections in any site with culture results showing CRE as etiologic agents.

#### Definition of infection

Healthcare-associated infections were diagnosed according to Centers for Disease Control and Prevention definitions [16]. Severity of infections and sepsis were categorized as stated by the consensus meeting of the American College of Chest Physicians/Society of Critical Care Medicine criteria. Severe sepsis was defined as sepsis that was associated with tissue hypoperfusion and organ dysfunction that was depicted by oliguria, lactic acidosis, altered level of consciousness and hypotension without a need for vasopressors. Septic shock was indicated when administering vasopressors was necessary as a result of sepsis-induced hypoperfusion refractory to adequate fluid resuscitation [17].

The following demographic data were collected: age, gender, and the presence of comorbidities. Comorbidities were defined according to Charlson’s criteria and patients were categorized in four groups according to the number of comorbidities present (0, 1, 2, or ≥ 3 comorbidities) [18].

- The clinical variables were: admission to the intensive care unit (ICU), length of hospital and ICU stay, dialysis, site of infection, samples for culture (quantitative tracheal aspirate, blood, urine, tissues, and fluids), presence of severe sepsis and septic shock, co-infection by other pathogens, colistin resistance, and death associated with infection. Only the first sample was considered when the patient had more than one infection. The adopted cut-off point for positivity of quantitative tracheal aspirate was 10^5^ CFU/mL. Only the antimicrobials initiated in the first 72 h after diagnose of ECR infections and used for more than 48 h were assessed. Initial therapy was categorized as the following: monotherapy, combination therapy, and the starting time (less than 12 h; between 12 and 24 h; between 24 and 72 h; after 72 h). Antimicrobial therapy was considered appropriate when given at least one in vitro sensitive drug for a minimum of 48 h. The loading dose of colistin and double dose of tigecycline were also evaluated. Infection-related mortality was considered when the cause of death was attributable to the infection and the event occurred during the treatment. Hospital mortality considered death from any cause measured at hospital discharge.

### Microbiology and molecular analysis

The bacteria were identified using the API 20E and API 20NE miniaturized method (6.0 version, bio-Mérieux, Marcy L’Etoile, France).

### Susceptibility testing

Minimum inhibitory concentrations (MICs) of colistin (USP-Reference Standard Colistin Sulfate), imipenem, meropenem, ertapenem, gentamicin, amikacin, tigecycline, and meropenem (Astra Zeneca) against the clinical isolates were determined using the broth microdilution method according to the Clinical and Laboratory Standards Institute (CLSI) protocol [19]. *K. pneumoniae* ATCC 13883 and *Escherichia coli* ATCC 25922, obtained from the American Type Culture Collection, were used as controls.

The MIC50 and MIC90 were determined and interpreted as follows: colistin according to the European Committee on Antimicrobial Susceptibility Testing (EUCAST) [20], tigecycline according to the Food and Drug Administration (FDA), and for all the other microorganisms, CLSI criteria was used [16].

### Polymerase chain reaction

PCRs multiplex for genes encoding carbapenemases (*bla*_OXA-48-*like*_; *bla*_NDM_; *bla*_KPC_) were performed in all isolates as previously described by Bradford, Chen, and Monteiro [21–23]. These three carbapenemases were chosen to be analyzed because they are the most frequent carbapenemases described in Brazil [23] and are among the most common types described around the world.

### Pulsed-field gel electrophoresis

PFGE was performed using Spe-I (Thermo Scientific, Sinapse Biotecnologia Ltda) digestion of chromosomal DNA Ultrapure Agarose (Invitrogen™, Life technologies) [24]. Restriction fragments were obtained by separation using a CHEF DR®III system (Bio-Rad, Hercules, Calif., USA). Patterns were interpreted using BioNumerics**®** version 7.1 (Applied Maths).

### Statistical analysis

A database was built using the Epi Info 7.1.5.2 program. A descriptive analysis was made of patient characteristics. Continuous variables were expressed as mean and standard deviation (SD), median and interquartile range (ITQ), and compared by Wilcoxon’s test; the categorical variables by Chi-squared and Fisher’s Exact tests. The outcome studied was death related to infection. The level of significance adopted for comparison of the variables in the bivariate test was the value of *p* < 0.05. A multivariate analysis using multiple logistic regression was performed to evaluate potential factors associated with death related to infection. Two cohorts were analyzed with the logistic regression, firstly all CRE infections were included in the first model and secondly CRE infections excluding UTIs were analyzed in the second model. Results are expressed as relative risk (RR) and 95 % confidence interval (CI). The variables with *p* < 0.10 in the bivariate analysis and biological plausibility were tested in the multivariate analysis by forward stepwise using two models, one with all infections and another excluding UTI infections.

## Results

During the study period, 127 patients developed infections caused by CRE, all healthcare-associated infections. The mean age was 55.7 (±18.0) years, 44.9 % aged above 60 years, and 88 (69.3 %) were male. One hundred and thirteen (89.0 %) isolates were *Klebsiella pneumoniae* and 8 were *Enterobacter* spp. Others pathogens found were 3 *Escherichia coli*, 1 *Citrobacter*, 1 *Serratia* and 1 *Providencia spp*. Fifty-five infections (43.3 %) were polymicrobial, and 36 (28.3 %) patients were co-infected with *Acinetobacter baumannii* (Table 1). All causes mortality at hospital discharge was 61.4 %. Infection-related mortality was 34.6 % and 30-day mortality was 28.3 % (Table 2).

**Table 1:** Univariate analysis of risk factors for infection-related death in 127 patients with infections caused by CRE

Characteristics	Total (*n* = 127)	Survivors (*n* = 83)	Deaths (*n* = 44)	RR (95 % CI)	P
Patient related					
Age (years) (SD)	55.7(18)				
Age over 60 (%)	57(44.9)	32(38.5)	25(56.8)	1.29(0.99–1.70)	0.03
Male (%)	88(69.3)	59(71.0)	29(65.9)	0.91(0.68–1.22)	0.34
ICU (%)	98(77.2)	60(72.3)	38(86.4)	1.29(1.01–1.65)	0.054
Comorbidities (%)	97(76.4)	67(73.6)	30(83.3)	1.39(1.10–1.75)	0.01
=1	23(18.1)	19(22.9)	4(9,0)	0.74(0.58–0.94)	0.04
=2	32(25.2)	17(20.5)	15(34.0)	1.30(0.92–1.85)	0.07
3 or more	42(33.0)	22(26.5)	20(45.4)	1.37(0.99–1.88)	0.02
Surgery (%)	59(46.5)	41(49.4)	18(40.9)	0.88(0.69–1.14)	0.23
Dialyses (%)	45(35.4)	18(21.7)	27(61.4)	1.98(1.36–2.88)	0.0001
Previous colonization (%)	76(59.8)	50(60.2)	26(59.0)	0.98(0.75–1.27)	0.52
Infection (%)					
Pneumonia	52(41.0)	25(30.1)	27(61.4)	1.60(1.18–2.18)	0.0006
UTI	51(40.2)	39(47.0)	12(27.3)	0.75(0.59–0.96)	0.02
Bloodstream	9(7.0)	7(8.4)	2(4.5)	0.82(0.56–1.20)	0.33
Tissue	13(10.2)	10(12.0)	3(6.8)	0.83(0.59–1.15)	0.27
Abdominal	2(1.6)	2(2.4)	0	0.64(0.56–0.73)	0.42
Sepsis	28(22.0)	27(32.5)	1(2.3)	0.58(0.48–0.70)	0.0001
Severe sepsis	21(16.5)	17(20.5)	4(9.0)	0.76(0.59–0.99)	0.07
Shock	63(49.6)	25(30.1)	38(86.4)	2.28(1.66–3.12)	0.0001
Infection site (%)					
Lungs	46(36.2)	23(27.7)	23(52.7)	1.48(1.07–2.03)	0.005
Urine	51(40.2)	39(47.0)	12(27.3)	0.75(0.59–0.96)	0.02
Blood	21(16.5)	11(13.2)	10(22.7)	1.29(0.84–1.99)	0.13
Tissue	11 (8.7)	7(8.4)	4(9.1)	1.02(0.64–1.64)	0.56
Others	8(8.0)	8(9.6)	0	0.63(0.54–0.72)	0.02
Pathogen (%)					
*K. pneumoniae*	113(89.0)	74(89.2)	39(88.6)	0.98(0.64–1.48)	0.57
Polymyxin-resistant	27(21.3)	17(20.5)	10(22.7)	1.04(0.75–1.44)	0.46
Polymicrobial	55(43.3)	34(41.0)	21(47.7)	1.10(0.84–1.42)	0.29
Co-infection *A. baumannii*	36(28.3)	22(26.5)	14(31.8)	1.09(0.81–1.47)	0.33
Co-infection *P. aeruginosa*	13(10.2)	7(8.4)	6 (13.6)	1.23(0.73–2.08)	0.26
bla_KPC_+	96(75.6)	68(81.9)	28(63.6)	0.68(0.46–1.00)	0.02

*CRE* carbapenem-resistant *Enterobacteriaceae*, *RR* relative risk, *CI* confidence interval, *SD* Standard deviation, *LOS* length of stay, *IQR* interquartile range, *UTI* urinary tract infection

**Table 2:** Comparison of mortality rates among 127 patients infected by carbapenem-resistant *Enterobacteriaceae* in the ICU and in the ward

Mortality	Overall (*n* = 127)	ICU (*n* = 98)	Ward (*n* = 29)	P
Hospital	78(61.4 %)	69(70.4 %)	9(31.0 %)	<0.001
Infection-related	44(34.6 %)	38(38.8 %)	6(20.7 %)	0.05
30 days	36(28.3 %)	30(30.6 %)	6(20.7 %)	0.21

*ICU* intensive care unit

Pneumonia and urinary tract infections were the most frequent sites of infection, with 52 (42 %) and 51 (40.2 %) cases, respectively. Most infections (77.2 %) occurred in patients in the ICU, and in this group, the mean APACHE II score was 20.6 (SD: 7.1), median SOFA at admission was 7.5 (IQR: 5-11), and median SOFA at discharge was 7.1 (IQR: 2.5-12). All patients with pneumonia were on mechanical ventilation. Median length of stay in the ICU was 18 (IQR: 8-28.5) days. Regarding the severity of the infection, 22.0 %, 16.5 %, and 49.6 % had sepsis, severe sepsis, and septic shock, respectively.

Comorbidities were present in 76.4 % of the cases, and the presence of three or more comorbidities was a risk factor for death in the bivariate analysis (RR: 1:37; 95 % CI: 0.99-1.88; *p* = 0.02). Fifty-nine (46.5 %) patients underwent surgery, and 35.4 % were submitted to dialysis. Dialysis (RR: 1.98; 95 % CI: 1.36-2.88; *p* <0.0001), pneumonia (RR 1.60, 95 % CI: 1.18-2.18; *p* <0.001), and shock (RR 2.28, 95 % CI: 1.66-3.12; *p* = <0.001) were risk factors for infection-related death, and UTI was a protective factor (RR 0.75, 95 % CI: 0.59-0.96; *p* = 0.02). The *Bla*_KPC_ gene was found in 75.6 %. Resistance to colistin occurred in 21.3 %, with no significant difference between deaths related or not to infection (20.5 % and 22.7 %, respectively, *p* = 0.46) (Table 1).

**Fig. 1:**
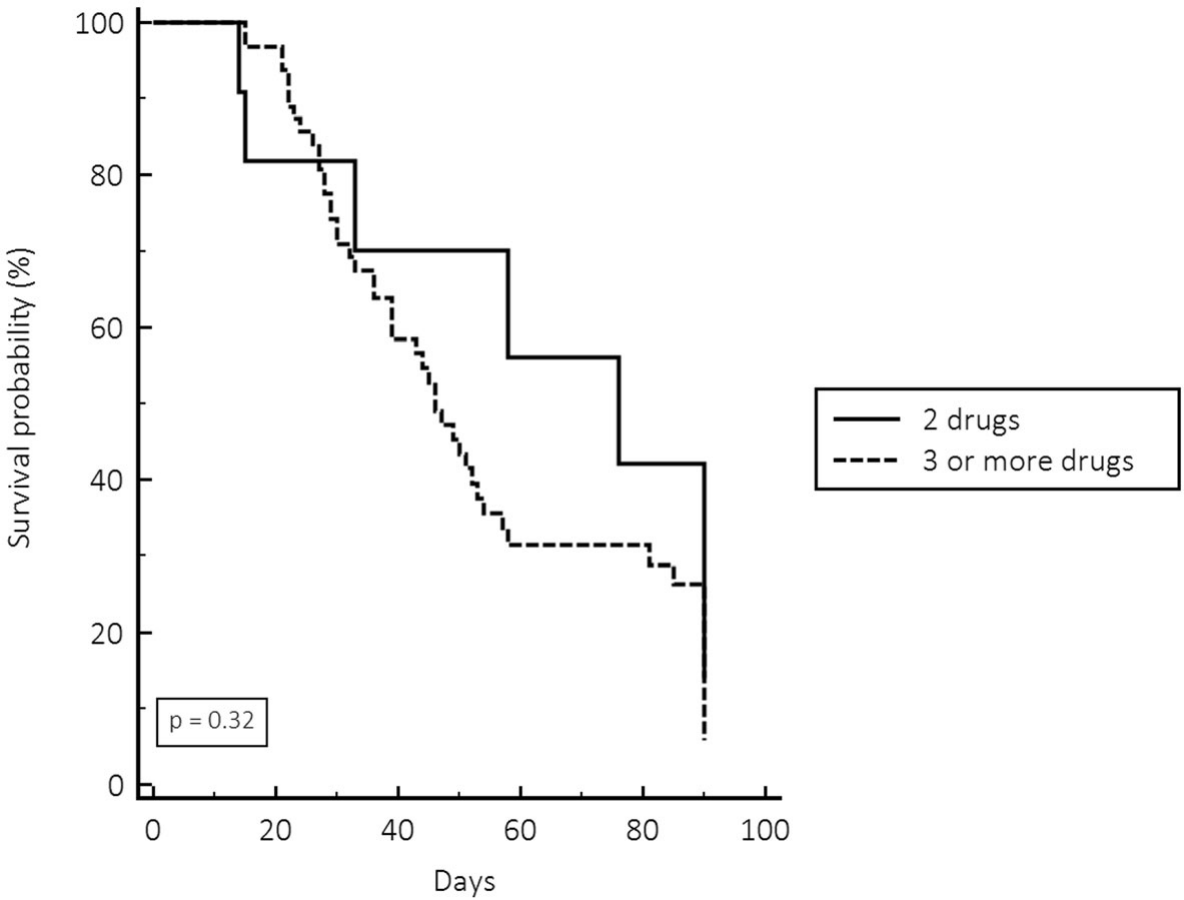
Kaplan Meier survival curve comparing combination therapy with two drugs or more than two drugs in the treatment of carbapenem-resistant infections, except urinary tract infections. Log rank test

Monotherapy was used in 29 patients (22.8 %), 28 (96.5 %) had UTI, and combination therapy comprising two, three, or more drugs was given to 98 (77.2 %) patients. Considering all infections except UTI, the use of two, three, or more drugs was not associated with differences in mortality, according to Kaplan Meier’s survival curve (Fig. 1). Twenty-seven patients had infection due to colistin-resistant CRE (colistin MIC = 4 – 64 μg/mL). The overall mortality in patients with UTI was 24 %; 6/28(21 %) patients that received monotherapy died compared with 6/23 (26 %) of patients that received combined therapy (*p* = 0.47). There was no difference in infection-related death comparing colistin-susceptible and colistin-resistant UTI (*p* = 0.41). Among eight patients with UTI caused by colistin-resistant CRE that received monotherapy, only one patient with high colistin MIC = 16 μg/mL evolved to death.

In the bivariate analysis, monotherapy was a protective factor for death related to infection (RR: 0.77, 95 % CI: 0.60-0.98, *p* = 0.05); however, most of the patients receiving monotherapy had UTI (Table 3).

**Table 3:** Univariate analysis of therapeutic regimens used in patients with carbapenem-resistant infections on infection-related deaths

Treatment	Total (*n* = 127) N (%)	Survivors (*n* = 83) N (%)	Deaths (*n* = 44) N (%)	RR (95 % CI)	P
Less than12 h onset	39 (30.7)	20 (24.1)	19 (43.2)	1.39 (1.00–1.94)	*0.02*
12 h onset and sensitive	31 (24.4)	14 (16.9)	17 (38.6)	1.59 (1.05–2.39)	*0.006*
Less than 24 h onset	53 (41.7)	30 (36.1)	23 (52.3)	1.26 (0.96–1.66)	0.059
Monotherapy	29 (22.8)	23 (27.7)	6 (13.6)	0.77 (0.60–0.98)	0.054
Combined therapy	98 (77.2)	60 (72.3)	38 (86.4)	1.29 (1.01–1.65)	0.054
2 drugs	22 (17.3)	16 (19.3)	6 (13.6)	0.87 (0.65–1.17)	0.29
3 drugs	53 (41.7)	31 (37.3)	22 (50.0)	1.20 (0.91–1.57)	0.11
4 drugs	23 (18.1)	13 (15.6)	10 (22.7)	1.19 (0.81–1.74)	0.22
Colistin	107 (8.2)	67 (80.7)	40 (90.9)	1.27 (0.98–1.66)	0.10
Loading dose of colistin	74 (69.2)	43 (64.2)	31 (77.5)	1.25 (0.94–1.66)	0.10
Tigecycline	69 (54.3)	43 (51.8)	26 (59.0)	1.10 (0.86–1.42)	0.27
Double dose of tigecyclin	46 (66.8)	29 (67.5)	17 (65.4)	0.96 (0.65–1.43)	0.53
Carbapenem	69 (54.3)	42 (50.6)	27 (61.4)	1.16 (0.90–1.49)	0.16
Aminoglycoside	82 (64.6)	51 (61.4)	31 (70.4)	1.14 (0.88–1.47)	0.20
Use of more two or more drugs with in vitro activity on isolates	71 (55.9)	45 (54.2)	26 (59.0)	1.07 (0.83–1.37)	0.36

*RR* relative risk, *CI* confidence interval

Sensitivity to colistin was observed in 78.7 % of the cases, to tigecycline in 58.3 %, and to gentamicin in 18.1 %. Only Fosfomycin showed great sensitivity in vitro (100 %), but its intravenous presentation is not available in the country of the research and it was tested in vitro only against polymyxin E-resistant strains (Table 4).

**Table 4:** In vitro antibiotic sensitivity profile using microdilution of 127 carbapenem-resistant *enterobacteriaceae* isolates

Antimicrobial	Sensitive %(n)	Tested number	MIC to sensitivity	Criteria
Polymyxin	78.7 (100)	127	≤2ug/mL	Eucast
Gentamicin	18.1 (23)	127	≤4ug/mL	CLSI
Tigecycline	58.3 (49)	84	≤1ug/mL	Eucast
	92.8 (78)	84	≤2ug/mL	FDA
Fosfomycin^a^	100.0 (29)	29	≤64ug/mL	CLSI
	96.5 (28)	29	≤32ug/mL	Eucast
Imipenem	11.0 (14)	127	≤1ug/mL	CLSI

*MIC* minimum inhibitory concentration, *Eucast* European committee on susceptibility testing, *CLSI* clinical and laboratory standards institute, *FDA*, food and drug administration

^a^Fosfomycin was tested only against polymyxin-resistant ERC

**Fig. 2:**
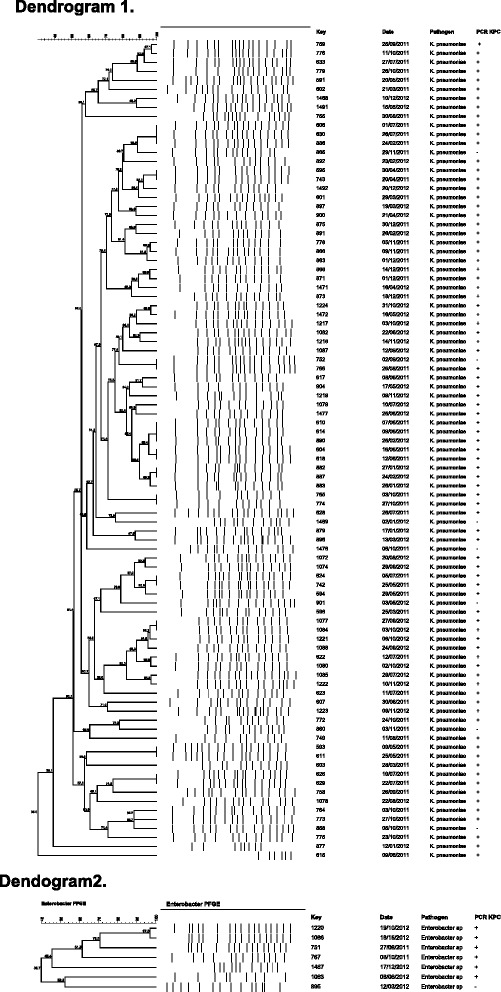
The dendrogram1 (113 *K. pneumoniae* infections) and dendrogram2 (7 *Enterobacter* spp) infections identified in the University Hospital of Londrina (Paraná, Brazil), from March 2011 to December 2012. PFGE = Pulsed Field Gel Electrophoresis; PCR: Polymerase Chain Reaction; KPC; *Klebsiella pneumoniae* carbapenemase

In the first multivariate analysis including all CRE infections, the presence of shock and dialysis remained as predictors of death related to infection in the first model (Table 5). In the second multivariate analysis, using a model that excluded patients with UTI, the presence of shock, old age, and dialysis remained as independent risk factors for death (Table 6). The dendrograms of 113 *K. pneumoniae* infections (Fig. 2) and 7 *Enterobacter* spp infections (Fig. 2) showed that they were polyclonal. We did not perform dendrogram of others pathogens because of number of isolates: 3 *Escherichia coli*, 1 *Citrobacter spp.*, 1 *Serratia spp.* and 1 *Providencia spp*.

**Table 5:** Regression analysis of risk factors associated with infection-related death among 127 patients infected by CRE between March 2011 and December 2012

Variable	Total	Survivors	Deaths	OR (95 % CI)	P
First model					
60 y or over	57 (44.9 %)	32 (38.5 %)	25 (56.8 %)	2.72 (0.93–7.95)	0.06
Shock	63 (49.6 %)	25 (30.1 %)	38 (86.4 %)	10.26 (2.95–35.68)	*0.0002*
Dialysis	45 (35.4 %)	18 (21.7 %)	27 (61.4 %)	2.94 (1.00–8.62)	0.04
Sensitive ATM less than 12 h	31 (24.4 %)	14 (16.9 %)	17 (38.6 %)	1.80 (0.63–5.14)	0.27
UTI	51 (40.2 %)	39 (47.0 %)	12 (27.3 %)	0.71 (0.14–3.62)	0.68
Pneumonia	52 (41.0 %)	25 (30.1 %)	27 (61.4 %)	1.58 (0.40–6.18)	0.50
Monotherapy	29 (22.8 %)	23 (27.7 %)	6 (13.6 %)	3.93 (0.71–21.53)	0.11
More than two comorbidities	42 (33.0 %)	22 (26.5 %)	20 (45.4 %)	1.27 (0.45–3.54)	0.64

*CRE* carbapenem-resistant infection, *OR* odds ratio, *CI* confidence interval, *ATM* antimicrobial

**Table 6:** Regression analysis models of risk factors associated with infection-related death among 76 patients infected by CRE, excluding urinary tract infections, between March 2011 and December 2012

Variable	Total	Survivors	Deaths	OR (95 % CI)	P
First model					
60 y or over	32 (42.1 %)	15 (34.0 %)	17 (53.1 %)	3.10 (0.81–11.87)	0.09
Shock	47 (61.8 %)	18 (38.3 %)	29 (61.7 %)	5.84 (1.30–26.12)	*0.02*
Dialysis	35 (46.0 %)	29 (61.7 %)	29 (61.7 %)	4.76 (1.22–18.55)	*0.02*
Sensitive ATM less than 12 h	25 (32.9 %)	11 (44.0 %)	14 (56.0 %)	1.51 (0.45–5.01)	0.49
Pneumonia	52 (68.4 %)	25 (48.0 %)	27 (56.0 %)	1.55 (0.40–6.03)	0.52
Monotherapy	1 (1.3 %)	1 (100 %)	0 (0 %)	0.0001 (0.00–0.00)	0.97
More than two comorbidities				1.50 (0.44–5.08)	0.50

*CRE* carbapenem-resistant infection, *OR* odds ratio, *CI* confidence interval, *ATM* antimicrobial

## Discussion

The present study described a prospective cohort of hospitalized adult patients with infection caused by CRE. The *bla*_KPC_ gene was the only one identified in the strains studied. Monotherapy was used for UTI, and combination therapy for other sites of infection. There was no difference in the Kaplan Meier survival curve comparing therapy with two, three, or more drugs to treat systemic infections. Age, dialysis, and presence of shock were independent risk factors for infection-related death.

The high mortality of 34 % observed in this study was similar to previous reports that showed mortality caused by *K. pneumoniae* bacteremia harboring KPC ranging from 39 to 82 % [2, 3, 9, 25]. In our study, 61.4 % of patients with pneumonia evolved to death; all patients where on mechanical ventilation in the ICU. Some studies have reported increased mortality among ICU patients [6, 26]. Qureschi et al. [25] described that 10 of 41 patients with pneumonia caused by *K. pneumoniae* harboring KPC evolved for death. Capone et al. [12] reported a 42.9 % mortality rate in patients with VAP caused by *K. pneumoniae* harboring KPC. More recently, Tumbarello et al. [27] described a large cohort study describing lower 14-day mortality among non-bacteremic patients (24,3 %) comparing to bacteremic (38,7 %). Among non-bacteremic patients, 85 patients had lower respiratory tract infections with overall mortality of 40 %. Twenty-five percent of patients with lower respiratory tract infections that received combined therapy evolved for death comparing with 49 % that received monotherapy (*p* = 0.03). Our patients had a mortality higher than the non bacteremic patients described by that author, but our study was composed mainly by ICU patients, compared to only 29 % ICU admissions by Tumbarello et al. [27].

In this study, bivariate analysis identified age, comorbidities, pneumonia, dialysis, and presence of shock as significant risk factors for mortality as well as the APACHE II and SOFA scores at ICU discharge. In the regression analysis the independent risk factors were related to the seriousness of patients’ conditions and age. Zarkotou et al. [28] and Correa et al*.* [8] also described APACHE II and age as risk factors for mortality and in the latter, dialysis and use of vasoactive drugs remained as independent risk factors in the multivariate analysis.

Several studies evaluated therapeutic regimens in attempts to treat these infections. Capone et al. [12] mentioned 23 different treatment regimens in a multicenter prospective study with 97 patients. The evaluation of multiple schemes is a complicating factor in those analyzes. In our study between monotherapy and combination therapy, we detected 16 different treatment regimens. Some studies pointed towards monotherapy as responsible for higher mortality, especially in systemic infections such as bacteremia [9, 15, 25]. In the present study there was no record of monotherapy being used to treat systemic infections, monotherapy was used to treat localized UTI with good response. There was neither difference in mortality in patients who began appropriate therapy within 12 h of the infection diagnosis nor benefit of using carbapenem in infections caused by CRE presenting MIC < 4 ug/mL to treated pneumonia. These findings differ from previous authors that demonstrated that carbapenem combination therapy could be useful in treating infections caused by low carbapenem MIC isolates [29]. The Kaplan-Meier survival curve showed no significant difference in mortality of systemic infections, mainly pneumonia, caused by CRE treated with combinations with two, three, or more drugs. These findings alert to the importance of improving knowledge regarding the use of combination therapy with more than two drugs to treat CRE pneumonia and UTI, thus, this strategy can increase costs and side effects. Several countries have described the polyclonal spread of KPC-producing CRE [3], similar to the findings of this study. Although, the study was performed in a single center, the polyclonality allowed us to show that our isolates demonstrated a high sensitivity to fosfomycin and tigecycline using FDA criteria, but low sensitivity using EUCAST criteria. On the other, the isolates showed high resistance to amikacin.

In the present study, the proportion of colistin-resistant infections was similar to what was previous reported by Zarkotou et al. [28] and Capone et al. [12], which demonstrated 25 to 37 % of resistance to colistin. However, unlike other studies, our data show no difference in mortality comparing patients infected by colistin-susceptible CRE with those with infections caused by colistin-resistant isolates. Among eight patients with UTI caused by colistin-resistant CRE that received monotherapy, only one patient with high colistin MIC = 16 μg/mL evolved to death. This could perhaps be explained by the site of infection mainly pneumonia and UTI. Previous authors have demonstrated the success of monotherapy in treating UTI caused by CRE [28, 30].

This study has limitations: it was conducted in a single center and the number of combination therapies used hinders the analysis of the benefit of a specific combination.

## Conclusion

CRE infections are polyclonal, have high overall and infection-related mortality, and KPC was the only carbapenemase identified in our isolates. Mortality was higher among patients with pneumonia. There was no significant difference in mortality comparing colistin-susceptible with colistin-resistant infections. The Kaplan-Meier survival curve showed no significant difference comparing combination therapy with two, three, or more drugs to treat systemic infections, mainly pneumonia. The independent risk factors associated with death were age, dialysis, and shock.

## Abbreviations

APACHE: Acute physiology and chronic health disease classification system
BSI: Bloodstream infection
CI: Confidence interval
CRE: Carbapenem-resistant *Enterobacteriaceae*
ICU: Intensive care unit
ITQ: Interquartile
KPC: Klebsiella pneumoniae carbapenemase
MIC: Minimum inhibitory concentrations
RR: Relative risk
SD: Standard deviation
SOFA: Sepsis-related organ failure assessment
UTI: Urinary tract infection

## References

[1] Adler A, Carmeli Y. Dissemination of the *Klebsiella pneumoniae* carbapenemase in the health care settings: tracking the trails of an elusive offender. MBio. 2011;2(6):10,1128;mBio.00280,11 10.1128/mBio.00280-11 PMC3324114 22186612

[2] Nordmann P, Cuzon G, Naas T (2009). The real threat of *Klebsiella pneumonia* carbapenemase-producing bacteria. Lancet Infect Dis.

[3] Munoz-Price LS, Poirel L, Bonomo RA (2013). Clinical epidemiology of the global expansion of *Klebsiella pneumoniae* carbapenemases. Lancet Infect Dis.

[4] Daikos GL, Tsaousi S, Tzouvelekis S (2014). Carbapenemase-producing *Klebsiella pneumoniae* bloodstream infections: lowering mortality by antibiotic combination schemes and the role of carbapenems. Antimicrob Agents Chemother.

[5] Bulik CC, Nicolau DP (2011). Double-carbapenem therapy for carbapenemase- producing *Klebsiella pneumoniae*. Antimicrob Agents Chemother.

[6] Marchaim D, Chopra T, Perez F (2011). Outcomes and genetic relatedness of carbapenem-resistant *enterobacteriaceae* at Detroit Medical Center. Infect Control Hosp Epidemiol.

[7] Mezzatesta ML, Gona F, Caio C (2011). Outbreak of KPC-3-producing and colistin-resistant, *Klebsiella pneumoniae* infections in two Sicilian Hospitals. Clin Microbiol Infect.

[8] Correa L, Martino MDV, Siqueira I (2013). A hospital-based matched case-control study to identify clinical outcome and risk factors associated with carbapenem-resistant *Klebsiella pneumoniae* infection. BMC Infect Dis.

[9] Kontopidou F, Giamarellou H, Katerelos P (2014). Infections caused by carbapenem-resistant *Klebsiella pneumoniae* among patients in intensive care in Greece: a multi-centre study on clinical outcome and therapeutic options. Clin Microbiol Infect.

[10] Grundmann H, Livermore DM, Giske CK, et al. Carbapenem non- susceptible *Enterobacteriaceae* in Europe: conclusions from a meeting of a national experts. Euro Surveill. 2010;15(46):1-13. 10.2807/ese.15.46.19711-en 21144429

[11] de Oliveira MS, de Assis DB, Freire MP (2015). Treatment of KPC-producing Enterobacteriacea: suboptimal efficacy of polymyxins. Clin Microbiol Infect.

[12] Capone A, Giannella M, Fortini D (2013). High rate of colistin resistance among patients with carbapenem-resistant *Klebsiella pneumoniae* infection accounts for an excess of mortality. Clin Microbiol Infect.

[13] Humphries RM, Keledesis T, Bard JD (2010). Successful treatment of pan-resistant *Klebsiella pneumoniae* pneumonia and bacteraemia with a combination of high dose tigecycline and colistin. J Med Microbiol.

[14] van Duin D, Kaye KS, Neuner EA, Bonomo RA (2013). Carbapenem-resistant Enterobacteriaceae: a review of treatment and outcomes. Diagn Microbiol Infect Dis.

[15] Lee GC, Burgess DS (2012). Treatment of *Klebsiella pneumoniae* carbapenemase (KPC) infections: a review of published case series and case reports. Ann Clin Microbiol Antimicrob.

[16] Horan T, Andrus M, Dudeck MA (2008). CDC/NHSN surveillance definition of health care-associated infection and criteria for specific types of infections in the acute care setting. Am J Infect Control.

[17] Levy MM, Fink MP, Marshall JC (2003). 2001 SCCM/ESICM/ACCP/ATS/SIS International Sepsis Definitions Conference. Crit Care Med.

[18] Charlson ME, Pompei P, Ales KL, MacKenzie CR (1987). A new method of classifying prognostic comorbidity in longitudinal studies: development and validation. J Chronic Dis.

[19] Institute CLS (2012). Performance Standards for Antimicrobial Susceptibility Testing; Nineteenth Informational Supplement, M100-S20.

[20] European Committee on Antimicrobial Susceptibility Testing (Eucast). 2012.

[21] Bradford PA, Bratu C, Urban M (2004). Emergence of carbapenem-resistant *Klebsiella* species possessing the class A carbapenem-hydrolyzing KPC-2 and inhibitor-resistant TEM-30 beta-lactases in New York City. Clin Infect Dis.

[22] Chen Y, Zhou Z, Jiang Y, Yu Y (2011). Emergence of NDM-1-producing *Acinetobacter baumannii* in China. J Antimicrob Chemother.

[23] Monteiro J, Widen RH, Pignatari ACC (2012). Rapid detection of carbapenemase genes by multiplex real time PCR. J Antimicrob Chemother.

[24] Pfaller MA, Isemberg HD (1993). Chromosomal restriction fragment analysis by pulsed-field gel electrophoresis. Clinical microbiology procedures handbook.

[25] Qureshi ZA, Paterson DL, Potoski BA (2012). Treatment outcome of bacteremia due to KPC-producing *Klebsiella pneumoniae*: superiority of combination antimicrobial regimens. Antimicrob Agents Chemother.

[26] Souli M, Galani I, Antoniadou A (2010). An outbreak of infection due to β-lactamase *Klebsiella pneumoniae* carbapenemase 2-producing *K. pneumoniae* in a Greek university hospital. Clin Infect Dis.

[27] Tumbarello M, Trecarichi EM, De Rosa FG (2015). Infections caused by KPC-producing Klebsiella pneumoniae: differences in therapy and mortality in a multicenter study. J Antimicrob Chemother.

[28] Zarkotou O, Pournaras S, Tselioti P (2011). Predictors of mortality in patients with bloodstream infections caused by KPC-producing *Klebsiella pneumoniae* and impact of appropriate antimicrobial treatment. Clin Microbiol Infect.

[29] Ceccarelli G, Falconi M, Giordano A (2013). Successful ertapenem-doripenem combination treatment of bacteremic ventilator-associated pneumonia due to colistin resistant KPC-producing *K. pneumoniae*. Antimicrob Agents Chemother.

[30] Cicora F, Mos F, Paz M, Allende NG, Roberti J (2013). Infections with blaKPC-2-producing Klebsiella pneumoniae in renal transplant patients: a retrospective study. Transplant Proc.

